# Wearable Wireless Tactile Display for Virtual Interactions with Soft Bodies

**DOI:** 10.3389/fbioe.2014.00031

**Published:** 2014-09-01

**Authors:** Gabriele Frediani, Daniele Mazzei, Danilo Emilio De Rossi, Federico Carpi

**Affiliations:** ^1^School of Engineering and Material Science, Queen Mary University of London, London, UK; ^2^Research Center “E. Piaggio”, University of Pisa, Pisa, Italy

**Keywords:** actuator, dielectric elastomer, display, electroactive polymer, soft, tactile, virtual, wearable

## Abstract

We describe here a wearable, wireless, compact, and lightweight tactile display, able to mechanically stimulate the fingertip of users, so as to simulate contact with soft bodies in virtual environments. The device was based on dielectric elastomer actuators, as high-performance electromechanically active polymers. The actuator was arranged at the user’s fingertip, integrated within a plastic case, which also hosted a compact high-voltage circuitry. A custom-made wireless control unit was arranged on the forearm and connected to the display via low-voltage leads. We present the structure of the device and a characterization of it, in terms of electromechanical response and stress relaxation. Furthermore, we present results of a psychophysical test aimed at assessing the ability of the system to generate different levels of force that can be perceived by users.

## Introduction

Human computer interfaces able to provide user with tactile feedback are spreading in several fields. For instance, tactile displays are used to allow users to interact with computer generated objects in virtual environments. Examples of application like training for medical operators (Champion et al., 2008), teleoperation (Sarakoglou et al., 2012), computer aided design (Liu et al., 2004), and 3D model exploration (Seth et al., 2011) might take advantage of interfaces able to mimic accurately and realistically the tactile feeling produced by the contact with a real object.

Several commercial tactile displays are currently available. For example, the grounded interface Geomagic Touch (Geomagic, Inc., USA) is accurate and can produce considerable forces, even though it is far from being portable and wearable (Prattichizzo et al., 2013). Glove-type interfaces made by CyberGlove Systems LLC (USA) are intended to improve the wearability of these devices. The hand-grounded CyberGrasp™ system can provide force feedback to the five fingers (Aiple and Schiele, 2013); however, its complex mechanics, made of tendons routed via an exoskeleton and the need for an external actuator module limit its portability (Prattichizzo et al., 2013). The CyberTouch™ system can produce complex tactile feedback patterns, although it works only in vibration mode (CyberGlove Systems, 2014).

Devices aimed at overcoming these limitations have been studied. For instance, Scilingo et al. (2010) proposed a grounded display of variable compliance aimed at integrating kinesthetic and tactile information. Minamizawa et al. (2007) presented the gravity grabber: a wearable and portable tactile display consisting of two motors fixed on the back of the finger and a belt able to apply force to the user’s finger pulp.

However, to the best of our knowledge, no tactile devices are currently available to mimic virtual contacts of a finger pulp with soft bodies, via soft interfaces. Such devices are needed for applications in virtual reality systems aimed at mimicking, for instance, interactions with biological tissues, for surgical training, e.g., palpation of soft tissues or robot-assisted minimally invasive surgery.

We believe that proper mimicking of tactile interactions with soft bodies requires not only a mechanism able to modulate the contact force, but also to deliver quasi-static forces via soft interfaces. This is aimed at ensuring that the deformable finger pulp conforms to the compliance of the soft body being explored. Moreover, devices conceived to that purpose should allow the user to freely move the hand and the arm while exploring the virtual soft body. Therefore, such tactile systems should be wearable and compact, and should have a light weight and a simple structure (with no gearings). They should also be acoustically silent and generate low heat, so as to favor comfort for the user. In order to overcome the typical inability of pneumatic and electromagnetic drives to meet all these requirements (Minamizawa et al., 2007; Scilingo et al., 2010), new actuation technologies are needed.

Here, we describe a new approach based on the technology known as dielectric elastomer actuators (DEAs) (Pelrine et al., 2000). Among the emerging field of the electromechanically active polymers (EAPs) DEAs represent one of the most promising technologies for soft actuation. They basically consist of a thin insulating elastomeric layer coated with two compliant electrodes, so as to form a deformable capacitor. When applying a voltage difference *V* between the two electrodes an electrostatic pressure is produced resulting in a thickness squeezing and consequent surface expansion (Bar-Cohen, 2004; Carpi and Smela, 2009).

With reference to a planar DEA unit, consisting of a dielectric elastomer layer, of thickness *d*, coated with complainant electrodes, the expression of this pressure in the direction orthogonal to the electrodes (i.e., parallel to the electric field *E*) is as follows (Pelrine et al., 2000):

(1) $$p=\varepsilon_{0}\varepsilon_{r}{\left(V∕d\right)}^{2}=\varepsilon_{0}\varepsilon_{r}E^{2}$$

where ε_0_ is the dielectric permittivity of vacuum and ε_r_ is the relative dielectric constant of the elastomer.

DEAs are particularly attractive as an electromechanical transduction technology to develop the tactile displays of interest here, as they are intrinsically soft and compact, and are characterized by low specific weight, silent operation and low power consumption (Pelrine et al., 2000; Brochu and Pei, 2010; Carpi et al., 2011a).

So far, to the best of our knowledge, the literature offers only one example of investigation on using DEAs for tactile displays aimed to be wearable. This refers to the work by Koo et al. (2006), who have experimented with an array of buckling multilayer DEAs, wrapped around the fingertip.

Here, we present a different architecture of a DEA-based wearable tactile display. The device was conceived using the technology known as hydrostatically coupled DEAs (HC-DEAs) (Carpi et al., 2010b). An incompressible fluid hydrostatically couples a DEA-based active part to a passive part interfaced to the user. In particular, the structure includes the following parts: an electromechanically active membrane, made of a DE film coated with compliant electrodes; an electromechanically passive membrane, working as the end effector in contact with the finger pulp; and an incompressible fluid contained between the two membranes, providing them with the shape of a spherical cap. Both membranes are radially constrained by bonding them to a stiff support.

**Figure 1:**
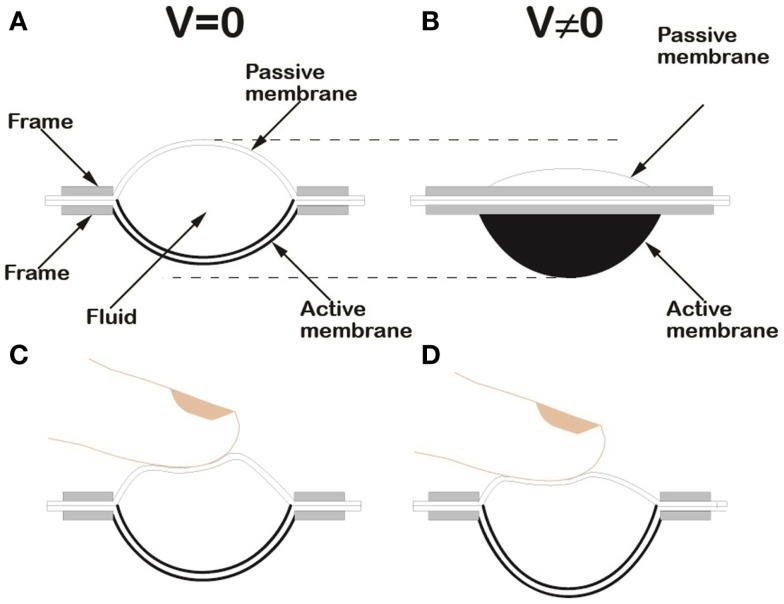
**Schematic drawing of the HC-DEA configuration used in this work**. Lateral section of the device in the rest state **(A)**. Lateral view of the device in an electrically induced state, due to an applied voltage difference *V* **(B)**. Loading of the passive membrane of a bubble-like HC-DEA: the internal redistribution of the fluid ensures that the active membrane keeps a uniform profile, both at rest **(C)** and when a voltage is applied **(D)**.

When a voltage is applied to the electrodes of the active membrane, the occurring surface expansion makes it to buckle outwards, owing to its pre-curvature. This effect induces the passive membrane to follow inwards, as the fluid’s volume is constant. So, a fluid-mediated hydrostatic transmission between the two membranes is established, as presented in Figure 1.

An electromechanical model of this type of actuators is presented in (Wang et al., 2012).

This principle allows for electrically safe transmission of actuation from the active membrane to the fingertip, without any direct contact between them (Figure 1).

Moreover, any distortion of the passive membrane introduced by the finger does not affect the shape of the active membrane (Figures 1C,D). Indeed, the redistribution of the fluid maintains a suitable uniformity of the active membrane’s curvature, changing its radius of curvature only. Such a self-compensation effect preserves the functionality of the device, which otherwise could be compromised.

The possibility to ensure a proper electrical insulation between the actuation part and the fingertip is of key importance, as any DEA requires today high driving voltages (usually of the order of 1 kV) (Brochu and Pei, 2010; Carpi et al., 2010a, 2011a). This is due to the low dielectric constant of state-of-the-art elastomers and the high thickness of the films resulting from their processing (Eq. 1). Implications are going to be specifically addressed in the Section “Discussion.”

**Figure 2:**
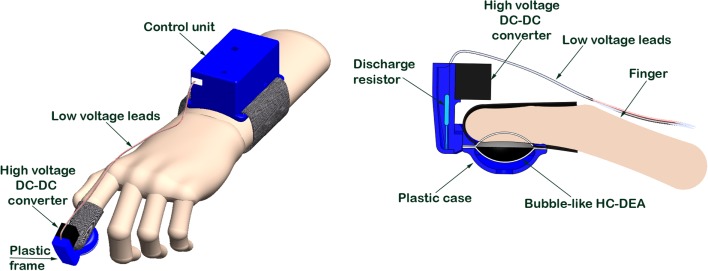
**Schematic drawings of the proposed fingertip wearable tactile display**.

In this work, the tactile display was conceived as a bubble-like HC-DEA integrated within a plastic case arranged at the fingertip, so as to have the finger pulp in contact with the passive membrane of the actuator, while the active membrane is protected by a plastic chamber. The structure of the device is shown in Figure 2. The specific materials and methods used are described in the next section.

## Materials and Methods

### Actuator manufacturing

**Figure 3:**
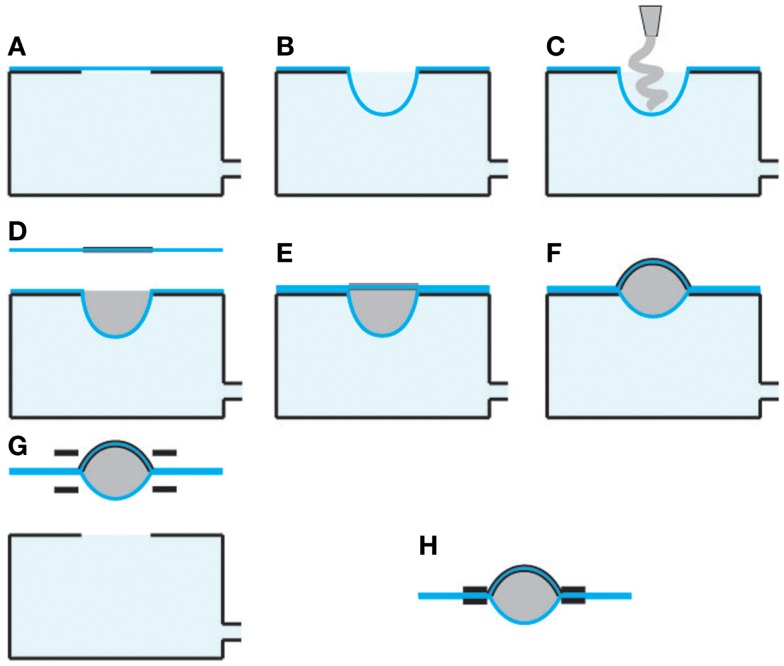
**Fabrication steps for a bubble-like HC-DEA**. The passive membrane is placed over an empty chamber having a circular hole **(A)**. Vacuum is applied in order to deform the membrane and create a cavity **(B)**. The cavity is filled with silicone grease **(C)**. The active membrane is coupled to the other membrane **(D)** and bonded to it **(E)**. Vacuum is released **(F)**. The membranes are removed from the vacuum chamber **(G)**, and bonded to a plastic frame **(H)**.

The bubble-like HC-DEA consisted of two membranes made of an acrylic elastomer film (VHB 4910, 3M, USA), bi-axially pre-stretched four times. The pre-stretch caused a reduction of the film thickness from 1 mm to 62.5 μm. One membrane was purely passive while the other was made active by coating both sides of it with carbon conductive grease (846, M.G. Chemicals, Canada), so as to obtain compliant electrodes. Each electrode had a circular shape and a radius of 10 mm. The pre-stretched passive membrane was placed over an empty chamber having a circular hole of the same size of the electrode. Vacuum was applied in order to deform the membrane and to create a cavity that was then filled with 1 ml of a dielectric silicone grease (8462, M.G. Chemicals, Canada). The active membrane was then coupled to the other membrane. The adhesiveness of the VHB film allowed for proper bonding. After 10 min the membranes were removed from the vacuum chamber, and bonded to a stiff plastic frame. Figure 3 shows the fabrication steps. The resulting final shape of each membrane was a spherical cap having a height of 7 mm and a base radius of 10 mm. The actuator was integrated within a plastic case, which was properly shaped so as to lodge the fingertip, allowing the pulp to be in contact with the passive membrane, as shown in Figure 2. The figure also shows that the plastic case hosted a miniaturized (about 1 cm^3^) high-voltage DC–DC converter (EMCO Q50, EMCO High Voltage, USA), used to drive the active membrane. The converter was fed with a 0–4.5 V signal to generate a 0–4.5 kV input for the actuator.

The plastic case also hosted a high-voltage discharge resistor of 50 MΩ, which was arranged in parallel to the actuator and had the following two functions. First, it allowed the converter to work with proper electrical loading. Second, it allowed the actuator to be discharged whenever the output of the converter had to be reduced.

In order to provide the user with a tactile feedback while interacting with a virtual body via a stimulation of the finger pulp with an electrically variable force, the converter was controlled by an external unit, as described below.

### Control hardware and software

A custom-made wireless control unit was developed. It was based on an Arduino FIO board, equipped with a 4.7 LiPo battery and a Bluetooth radio unit. Since the Arduino board was powered at 3.3 V, it was not able to directly drive the DC–DC high-voltage converter at 4.5 V. To overcome this limitation, the following driving circuit was developed. One of the pulse width modulation (PWM) pins of the Arduino board was used to drive a MOSFET transistor, which was connected to the LiPo battery. This extended the control signal range up to 4.7 V, i.e., above the maximum value required by the converter. This circuitry was arranged in a box on the forearm and connected to the converter via low-voltage leads (Figure 2).

Also, a firmware for the Arduino board was developed. It managed a wireless communication between the control unit and external Bluetooth devices working through virtual serial ports. Nowadays, serial ports are widespread and easy to control through various programing languages and operating systems.

A simple character-based protocol allowed us to control the driving voltage. In order to allow experimenters to easily use the system, a graphical user interface was developed using the.NET 4 c# programing language. It allowed for an easy configuration of the system and control of the actuation mode through virtual buttons. The character-based control protocol implemented would allow for a straightforward integration of the device in complex immersive environments like caves, and virtual reality spaces, where various systems are connected together typically using different communication protocols.

### Performance assessment

#### Free stroke and blocking force

**Figure 4:**
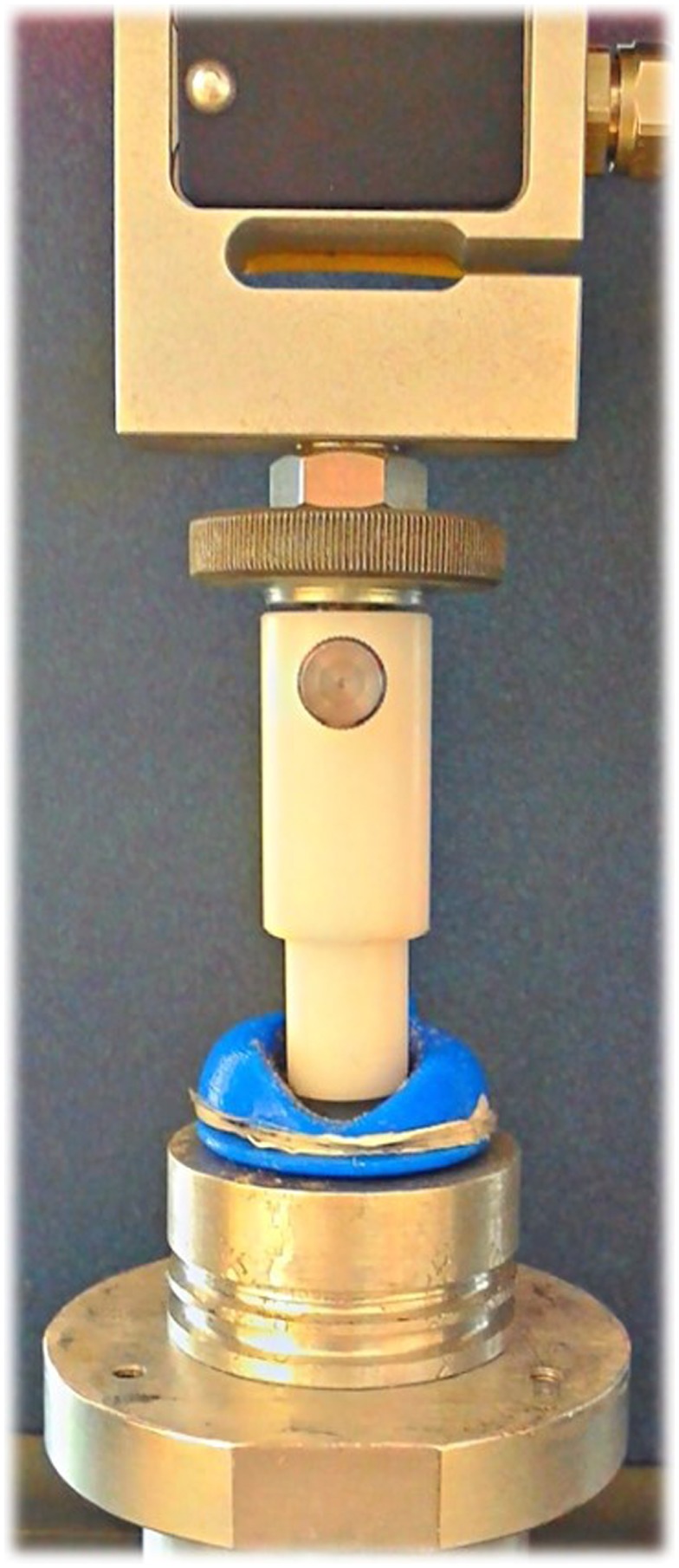
**Experimental setup for the mechanical and electromechanical characterization tests**.

Assessment of the static electromechanical performance of the tactile display was obtained by measuring free stroke and blocking force at the apex of the passive membrane. For different driving voltages, stroke and force were measured with a double-column dynamometer (Z005, Zwick Roell, Germany) according to the procedure described in Carpi et al. (2011b). In particular, a cylindrical indenter was mounted on the mobile crossbar of the machine, attached to a load cell. The indenter had a diameter of 12 mm, so as to fit with the internal part of the plastic case of the device. The crossbar was then moved, until the load cell detected a contact between the indenter and the actuator’s passive membrane, as shown in Figure 4. Then the actuator was electrically driven, so as to cause a displacement of the membrane’s apex. After that, the tool was brought in contact again with the actuator. The distance covered to restore contact was considered as the active displacement (free stroke). Subsequently, the voltage was turned off, without changing the position of the tool. As a result of this, the relaxation undergone by the actuator while trying to recover its rest shape generated a force, whose steady-state final value was considered as the blocking force. Finally, the crossbar was brought back to the initial position, and the actuator was allowed to fully recover its original shape. The test was repeated for voltages up to 4.5 kV, which was used as a safe limit to avoid electrical breakdown.

#### Blocking force with constant deformation

A blocking force test with constant deformation was also performed in order to take into account the fact that the finger pulp has to be held at a fixed position corresponding to a certain deformation of the actuator’s passive membrane. In order to estimate the force delivered in that condition, a test was performed as follows. While driving the actuator at the maximum voltage, the indenter was brought in contact with the displaced top membrane and maintained in that position during the entire test. This provided the actuator with a constant deformation aimed at reproducing the condition in which the finger impedes the passive membrane to fully recover its original shape. The blocking force was then measured at different voltages.

#### Stress relaxation

For the maximum voltage investigated, a stress relaxation test was performed as follows. The indenter was brought in contact with the electrically displaced membrane and maintained in that position for the entire test. After switching off the driving voltage, the force that the actuator generated while trying to recover its shape was measured for 10 min.

### Psychophysical test

In order to assess the ability of the system to generate different levels of force that can be distinguished by the user, the following psychophysical test was performed. Ten volunteers interacted via their fingertip with a virtual plane, and they were asked to report on the perceived force. This perceptual task was implemented as described below, for each of the five reference voltages V1–V5 corresponding to five equally spaced reference forces defined in the Section “Prototype Display and Electromechanical Performance.”

**Figure 5:**
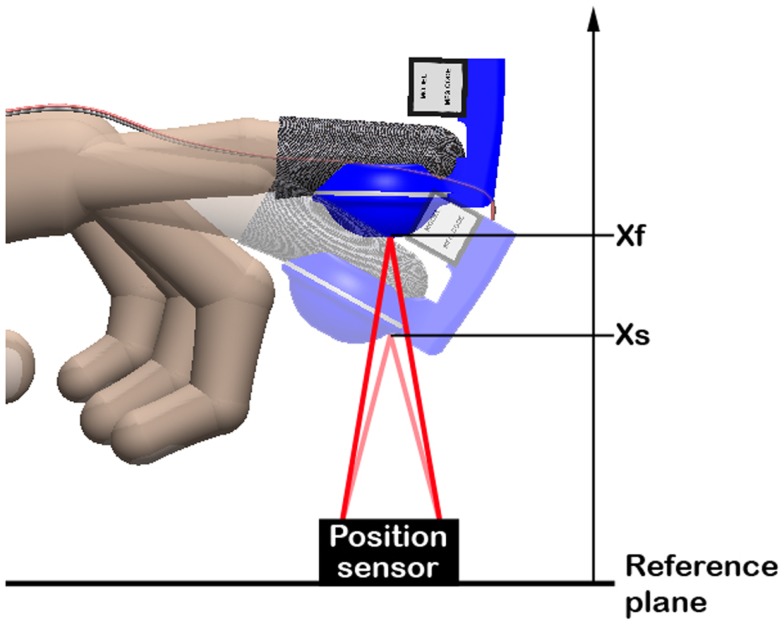
**Schematic representation of the movement that volunteers where asked to perform during the psychophysical test**.

The subject was asked to wear the tactile display on the dominant hand’s index finger. The subject was instructed to lift and lower the finger several times along the direction orthogonal to the plane, so as to reproduce the movement of probing a surface, as sketched in Figure 5.

The fingertip distance from the plane was continuously tracked by a position sensor (IR reflective distance sensor 10 cm, Phidgets Inc., Canada), as shown in Figure 5. Whenever the fingertip reached a certain distance *x*_s_, corresponding to the position of the virtual plane, the driving voltage of the tactile display was switched from 4.5 kV to one of the five reference voltages V1–V5. After the subject familiarized with the stimulus, the voltage was slowly increased, so as to slowly reduce the force. The subject, who kept probing the virtual plane, was asked to report when a difference with respect to the initial reference stimulus was perceived. The difference between the corresponding value of force and the reference force was then recorded. According to standards methods to characterize a perceptual threshold, this value represented the “just noticeable difference” (JND) (Falmagne, 1986).

This test was repeated by changing the reference voltage (for each voltage, the test was performed two times with each volunteer), obtaining JND values corresponding to the five reference stimuli.

## Results

### Prototype display and electromechanical performance

**Figure 6:**
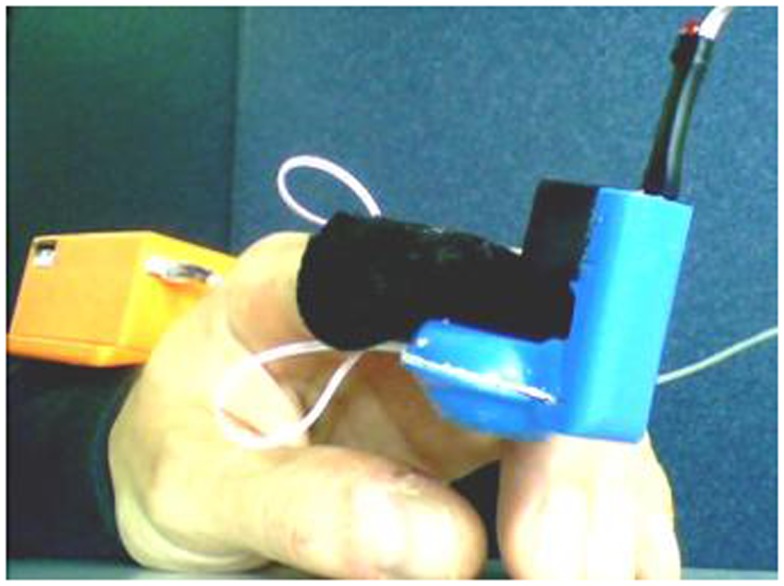
**Picture of the prototype fingertip display**. A box containing the wireless controller is visible in the rear part, arranged on the user’s forearm.

A prototype of the tactile display is shown in Figure 6.

**Figure 7:**
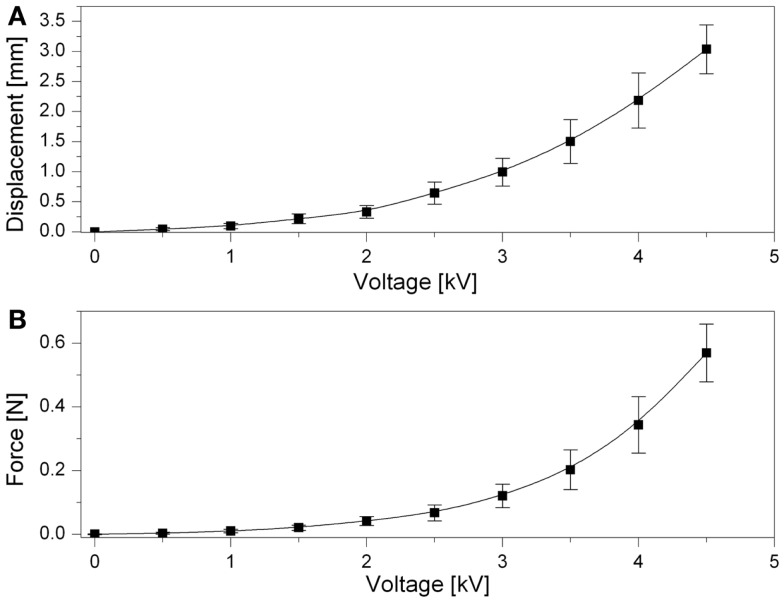
**Actuator’s performance**. Voltage-induced free stroke of the top passive membrane **(A)**. Blocking force versus voltage **(B)**. Error bars represent a 95% confidence interval. Fitting lines are used as a guide for the eye.

The voltage-induced response of the actuator is presented in Figure 7 in terms of displacement and force. The maximum displacement of the cap’s apex at 4.5 kV was about 3.25 mm while the active force was about 0.6 N.

**Figure 8:**
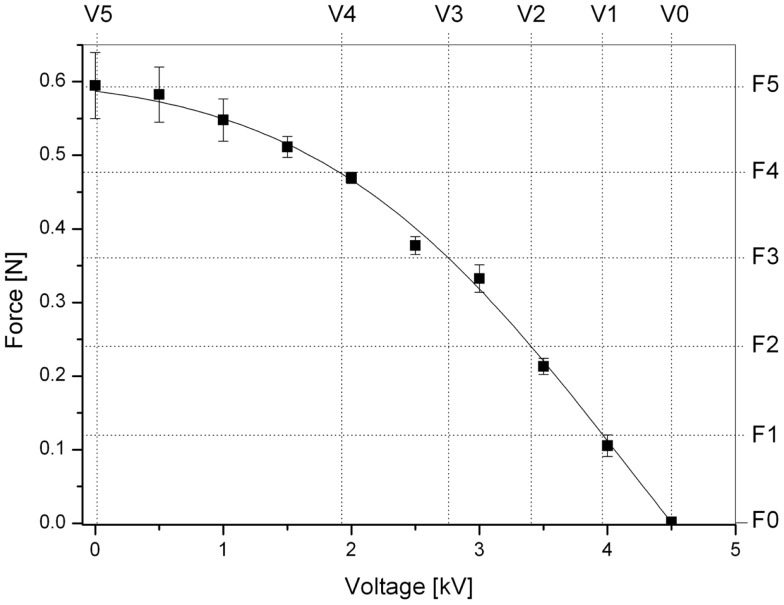
**Force perceived by the user as a function of the applied voltage while the finger is kept at a constant position corresponding to the maximum displacement (3.25 mm) that the actuator is capable of producing**. The five levels of force F1–F5 used for the psychophysical test are indicated. Error bars represent a 95% confidence interval. A fitting line of the experimental data is used as a guide for the eye.

Figure 8 shows the dependence of the blocking force on the voltage for a constant deformation equal to the maximum active displacement (3.25 mm) measured during the free-stroke test. So, this is the compressive force perceived by the user while the finger is kept at that constant position.

Figure 8 also shows the five equally spaced values of force (F1–F5) used during the perceptual test (F0 corresponds to a null force). Their values were extrapolated from the curve used to interpolate the experimental data.

### Viscoelastic performance

**Figure 9:**
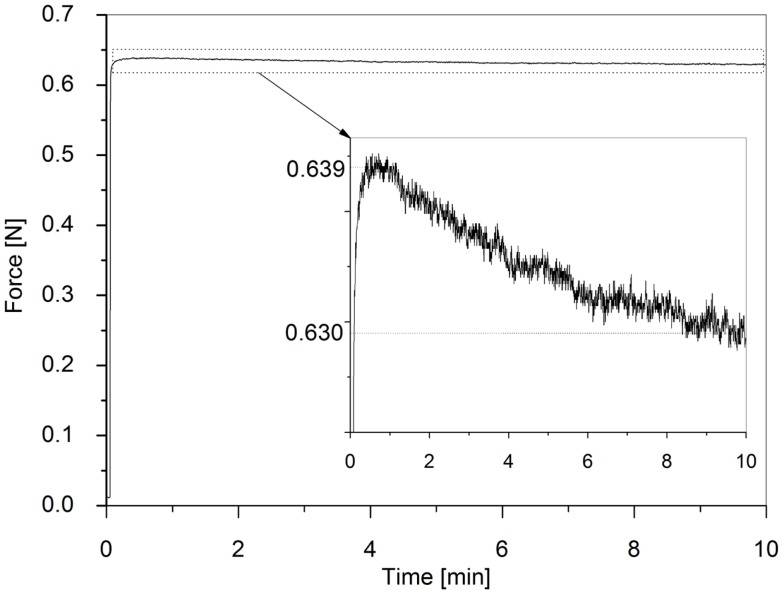
**Stress relaxation test over 10 min**. The inset shows a magnification.

Figure 9 presents the variation of the force exerted on the measurement tool during the stress relaxation test. The force was found to drop from 0.639 to 0.630 N in 10 min, corresponding to a relative variation of 1.4%.

### Psychophysical test

**Figure 10:**
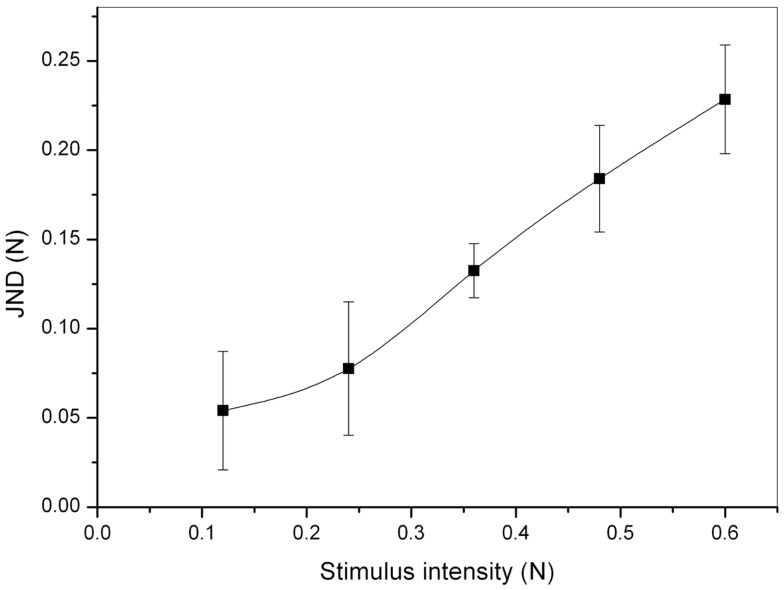
**Outcome of the psychophysical tests: JND as a function of the stimulus intensity (force)**. Error bars represent a 95% confidence interval.

Figure 10 reports results from the perceptual psychophysical tests. JND values are plotted as a function of the five equally spaced reference forces F1–F5 defined in Figure 8. As an overall measure of the ability of the device to generate small differences in stimuli that can be perceived by the user, the Weber constant (slope of the curve) was calculated from a linearization over the last four data points. It was found to be *k* = 0.4.

## Discussion

In this work, DEAs were used to mimic contact with a soft surface. This work moves from previous studies (Carpi et al., 2009, 2010b), where we had introduced HC-DEAs as a means to implement tactile displays. Here, we took a further leap, showing how to make them wearable and portable. The device was capable of mimicking a virtual contact with soft bodies via direct mechanical stimulation of the finger pulp.

While the demonstrated prototype was effective, both the display and the control unit were assembled using off-the-shelf materials (elastomer film) and electrical components (high-voltage converter and resistor, and microcontroller), certainly not optimized for this application. Custom-made materials and miniaturized electrical components might lead to increased performance and reduced encumbrance of the overall system, as well as increased wearability.

In particular, the dielectric elastomer used is one of the most studied commercially available materials for DEAs (Pelrine et al., 2000; Carpi et al., 2011a). Although it allows for significant electromechanical strains and stresses, it is not optimized to be used as a DEA material. It is well known to show a poor viscoelastic response, with significant creep and stress relaxation (Liu et al., 2014). Nevertheless, in this work we showed that using this material for the conceived quasi-static tactile display makes sense. Indeed, the viscoelastic test allowed us to assess that the tactile feedback (force) is practically not affected by relaxation for the targeted quasi-static conditions of operations, i.e., for applications that in general would require changing the displayed force let us say every second or more. However, depending on more specific application requirements, the actual effect of the viscoelastic losses on the device performance should be evaluated with *ad hoc* tests. Any specific requirement should be translated into a selection of elastomers with suitable electromechanical and viscoelastic properties.

The adopted elastomer film required high driving voltages. The need for dealing with voltages of the order of 1 kV is certainly a limitation. However, the generation of voltages so high is not particularly problematic from a technical standpoint, considering that there is no need for high driving powers (the loads are capacitive) and that all the high-voltage parts are insulated from the user. Indeed, the required voltage was generated with a voltage multiplier, which not only was compact but also allowed for a battery-operated circuitry. This allowed the system to be portable and relatively safe. The major drawback, when dealing with high voltage components, is represented by the costs, since high-voltage components are more difficult to miniaturize and have a relatively lower market, as compared to low-voltage units.

Significant research and industrial efforts are nowadays spent to produce new elastomer films that can exhibit suitable electromechanical responses at lower voltages. In particular, the synthesis of new materials with higher dielectric constant and the processing of thinner films are catalyzing much attention (Carpi et al., 2011a). The latter solution is likely to be achieved in the short term, considering that it has already been demonstrated to be viable, by several groups. Thinner films that can be driven at voltages as lows as few hundred volts would allow for using compact and low-cost circuitry currently adopted for piezoelectric actuators.

Furthermore, the DC–DC converter (not optimized for the application) introduced a significant loss of power. Indeed, as reported in the datasheet (EMCO High Voltage Corporation, 2014), at the maximum input voltage of 5 V, the input current was 250 mA, corresponding to an input power of 1.25 W. However, the output power was 0.5 W (5 kV at 100 μA), corresponding to an internal power loss of 0.75 W. Moreover, by realistically assuming that the actuator’s input resistance was greater than that of the external resistor (50 MΩ), and, so, by assuming that the converter’s output current was mostly absorbed by the resistor, the latter dissipated the greatest part of the output power.

Notwithstanding these electrical inefficiencies (requiring *ad hoc* components), the display’s mechanical response anyhow allowed us to provide users with tactile stimuli that could be properly perceived, as demonstrated by the psychophysical tests. Future developments might envisage different types of stimulation strategies, as the device is electrically tunable. As an example, the force could be finely modulated so as to mimic a progressive indentation of the finger within a virtual body.

In conclusion, while there is room for significant improvements in terms of materials and components, the described prototype showed the potential of the new technology proposed here. It paves the way for novel tactile displays able to simulate contact with virtual soft bodies via soft interfaces, while offering low weight, no acoustic noise, no heating, scalability, and low power consumption.

## Conflict of Interest Statement

The authors declare that the research was conducted in the absence of any commercial or financial relationships that could be construed as a potential conflict of interest.
